# Evaluation on soil fertility quality under biochar combined with nitrogen reduction

**DOI:** 10.1038/s41598-021-93200-0

**Published:** 2021-07-02

**Authors:** Xiaoqin Tian, Zhuo Li, Yifan Wang, Biao Li, Longchang Wang

**Affiliations:** 1 College of Agronomy and Biotechnology, Southwest University, Chongqing, 400715 China; 2 Crop Research Institute, Sichuan Academy of Agriculture Sciences, Chengdu, 610066 Sichuan China

**Keywords:** Ecosystem ecology, Ecology

## Abstract

A two-year consecutive field experiment was conducted in purple soil in southwest China, to clarify the effects of biochar (0, 10, 20 and 40 t ha^−1^, namely, B0, B10, B20 and B40) combined with nitrogen reduction (100%, 80% and 60% of conventional nitrogen application rate, namely, N100, N80 and N60) on soil fertility. The performance of thirty-four indices related to soil chemical, physical and biological properties was evaluated by gray correlation analysis, principal component analysis and cluster analysis to determine the most appropriate mode for soil fertilization, and to identify the main soil environmental factors affecting rapeseed yield under the biochar combined with nitrogen reduction. The results indicated that available phosphorus, geometric mean diameter of water stability, fungi number, and the utilization of sugars, amino acids, polymers and carboxylic acids by microorganisms could be used as the main soil factors affecting rapeseed yield. The highest score of soil quality was observed in N100B10 treatment, followed by N80B10 and N100B20 treatments, which were almost in line with the results of rapeseed yields. Cluster analysis classified 12 treatments into 5 main groups on the basis of the measured parameters, which was mostly consistent with the result of soil quality scores. Considering both economic and environmental benefits, 10 t ha^−1^ biochar combined with 144 kg ha^−1^ nitrogen was the best combination to restore crop productivity and soil quality, and to achieve nitrogen decreasing and benefit increasing. This study provided scientific basis for the rational fertilization and scientific management of biochar combined with nitrogen fertilizer in purple soil area of southwest China.

## Introduction

Soil fertility is the core of soil quality as well as the foundation of sustainable agricultural development, which plays an important role in maintaining soil quality and ensuring the sustainable use of soil resources^1^. In recent years, due to unreasonable fertilization management measures, especially over-reliance on nitrogen fertilizers in southwest China as main rapeseed producing area in China^2^, has caused many problems such as soil acidification, greenhouse gas emissions, groundwater nitrate pollution, organic matter content declining and production efficiency decreasing and so on^3–6^, which has become a serious threat to the sustainable development of agriculture in southwest China. Therefore, it is very urgent to explore economic and effective fertilization measures for achieving the sustainable development of agriculture.

Biochar is widely used in soil improvement as well as crop yield increase because of its unique structure and properties (such as abundant pores and large specific surface area)^7–10^. Liu et al.^11^ and Wang et al.^12^ showed that biochar significantly enhanced the soil quality, and crops yield and quality for the improvement of soil pH, activated carbon component, soil aggregation, total soil porosity and soil microbial biomass. Besides, the appropriate amount of nitrogen fertilizer plays an important role in increasing crop yields and soil quality. Nabilla et al.^13^ proved that urea (300 kg ha^−1^) gave the best black rice yield with high 1000-grain weight and high soil fertility. A number of previous studies also have proved that biochar combined with chemical fertilizer could reduce the leaching of soil nutrients, delay the release of nutrients, thereby increasing crop yields and improving fertilizer utilization^14–16^. Peng et al.^17^ suggested that biochar combined with chemical fertilizers could increase C stability and N retention in soil and improve N uptake by maize, while the loss of N was minimized. Moreover, Zaid et al.^18^ implied that within the appropriate nitrogen application range, the interaction of biochar and nitrogen might had an economical approach towards better utilization of nitrogen and sustainable crop production. However, because of the complexity of biochar, soil and crop characteristics and types, no a unanimous conclusion about biochar in soil improvement and crop productivity increasing. Zahra et al.^19^ indicated that there was an increase of 46.29% and 13.4% in grain yield and biological yield, respectively, due to the an increase of soil organic carbon in treatment containing biochar and compost over untreated control. However, Kalu et al.^20^ found that added biochars had minor long-term effects on the crop biomass yield, plant nutrient contents and plant nutrient uptake in both soil types (nutrient-poor, coarse textured Umbrisol and fertile, fine-textured Stagnosol). Therefore, biochar application in agricultural production should adapt to local conditions and be applied reasonably according to soil and crop types.

Purple soil is a unique and main soil type in southwest China. It is generally rich in calcium and nutrients, but is easily eroded and weathered for the shallow soil layer, so its physical and chemical properties, and biological characteristics are different from other varieties of soil^21^. Therefore, it is urgent to objectively and comprehensively evaluate the impact of biochar combined with nitrogen reduction on soil fertility, to find out the main soil environmental factors affecting rapeseed yield and to seek for the best combination ratio between biochar and nitrogen, so as to improve soil quality while improving economic benefits. Grey correlation degree, principal component analysis and cluster analysis are often used as statistical methods to assess soil fertility^22–23^. So far, there are few reports to comprehensively evaluate the effect of biochar combined with nitrogen reduction on purple soil quality from the physical, chemical and biological perspectives by the statistical methods. The aims of this study were to: (1) determine which soil environmental factors are the most important indexes affecting rapeseed yield under biochar combined with nitrogen reduction, and (2) identify the soil fertilization effect of biochar combined with nitrogen reduction to explore their best combined application amount. Results of this study will provide a scientific basis for the ration fertilization and scientific management of the combined application of biochar and nitrogen fertilizer in purple soil in southwest China.

## Materials and methods

### Research area

The study was conducted in the Yunyang Experimental Station (108° 54′ E, 30° 55′ N; altitude of 700 m), Southwest University, Chongqing, China. The study area has a subtropical monsoon humid climate with an average annual sunshine duration of 1500 h, average annual temperature of 18.4 °C average annual rainfall of 1100.1 mm, and the rain period predominantly prolongs from June to September. Local soil type is clay loam in texture and Dystric Purple-Udic Cambosols according to the Chinese Soil Taxonomy (CRGCST 2001). Basic properties of 0–20 cm soil layer were as follows: pH 7.29, total N 0.94 g kg^−1^, total C 7.14 g kg^−1^, available N 37.45 mg kg^−1^, available P 2.36 mg kg^−1^, and available K 72.58 mg kg^−1^, respectively.

The tested biochar was purchased from the Nanjing Qinfeng Straw Technology Co., Ltd. (Nanjing, China), which was made by pyrolysis of the rice (*Oryza sativa* L.) straw with limited oxygen supply at 500 °C for 2 h. Its properties were as follows: total N 0.61 g kg^−1^, total P 1.99 g kg^−1^, total K 27.15 g kg^−1^, total C 537.97 g kg^−1^ and pH 8.70.

### Experimental design

A two-year filed experiment (2017–2019) was performed in a completely randomized design with twelve treatments in triplicates including two factors. The first factor was the application of biochar including B0 (0 t ha^−1^), B10 (10 t ha^−1^), B20 (20 t ha^−1^) and B40 (40 t ha^−1^); and the second factor is the application level N fertilizer including conventional rate (application amount by local farmers)-180 kg N ha^−1^ (N100), 80% of conventional rate-144 kg N ha^−1^ (N80) and 60% of conventional rate-108 kg N ha^−1^ (N60). The plot size was 3 m × 6 m with a border (0.5 m wide) between plots. Biochar was applied to soil only in the first year before the sowing of rapeseed. Each treatment plot received the same amount of potassium (90 kg K_2_O ha^−1^) and phosphorus (90 kg P_2_O_5_ ha^−1^). Further details of fertilizer application have been reported by Tian et al.^24^, being the same for the two-year experiment. Weed, pesticide, and pest management kept the same with the local farmers’ rapeseed management practices. Winter rapeseed (Sanxiayou No.5) was used in the experiment, which was sowed on 21 October 2017 and on 16 October 2018, respectively, and was harvested on 1 May in both years (2018 and 2019).

### Sampling and analysis of soil and crop

#### Crop yield

Rapeseed was hand-harvested when 70–80% of total seeds changed their color from green to black on 1 May 2019, and each plot was separately harvested for seed yield. Seed yield was calculated using 6% as standard seed moisture content.

#### Soil indices

After the rapeseed harvest, soil samples were collected from all plots. Five sampling points were randomly selected within each plot. At each point, twenty soil cores of 2.5 cm diameter and 20.0 cm depth were taken in a 1 m radius of the point. All soil cores from each point were put in a plastic bag and thoroughly bulked, crumbled and mixed for physical, chemical and biological analyses. By dividing each soil sample into two subsamples, one subsample was ground, passed through a 2-mm sieve and was air-dried for the analyses of soil organic matter (SOM), total nitrogen (TN), total phosphorus (TP), total potassium (TK), alkali-hydrolyzale nitrogen (AN), available phosphorus (AP), available potassium (AK)^25^, particulate organic carbon (POC), water-soluble organic carbon (DOC), easily oxidized organic carbon (AOC)^26^, sucrase (SUC) and urease (URE)^27^, and another one was ground, passed through a 2-mm sieve and was stored in a refrigerator at − 20 °C for the analyses of structural and functional characteristics of soil microbial community^28^. At the same time, mixed soil samples (0–20 cm) from five points in each plot were taken using a shovel for soil aggregates analyses^24^.

Drying method was used to determine soil water content (SWC); soil temperature (ST) was measured by temperature probe on the LI6400–09 (LI-COR Inc., Lincoln, NE); potassium dichromate oxidation method was used to determine SOM and DOC content; TN was measured by the Kjeldahl method; TP was determined by Mo-Sb colorimetric method; TK was determined by NaOH melting and analyzed using an atomic spectrophotometry; AN was determined by diffusion-absorption method; AP was quantified by colorimetric analysis following extraction of soil with 0.5 mol L^−1^ NaHCO_3_; AK was measured using 1.0 mol L^−1^ CH_3_COONH_4_ extraction; POC was determined by sodium hexametaphosphate dispersion method; AOC was measured by potassium permanganate oxidation method; SUC was measured by 3,5-dinitrosalicylic acid colorimetric determination method; URE was measured by phenol-sodium hypochlorite indophenol colorimetry method; amount of bacteria (B), fungi (F), actinomycetes (A), gram-positive bacteria (GP), gram-negative bacteria (GN) was measured by the Bligh–Dyer method; utilization of sugars (S), amino acids (AA), phenolic acids (PA), carboxylic acids (CA), amines (AM) and polymers (P) by microorganism was measured using commercial Biolog EcoPlate (Biolog Inc., CA, USA).

Shannon index (H), Simpson index (D), and evenness index (E) were calculated by the following equations:

$$ {\text{AWCD}} = \sum {(C_{i}  - R_{i} )} /n $$

$$ {\text{H}} =  - \sum {P_{i} } (\ln P_{i} )\quad P_{i}  = (C_{i}  - R_{i} )/\sum {(C_{i}  - R_{i} } ) $$

$$ {\text{D}} = 1 - \sum P _{i}^{2} $$

$$ {\text{E}} = {\text{H}}/\ln {\text{S}} $$

where *n* is the 31 carbon sources on the ECO board; *C*_*i*_ and *R*_*i*_ and are the optical density values of the microwell and the control well respectively; *P*_*i*_ is the ratio of the absorbance of a particular well i to the sums of absorbance of all 31well at 120 h; S is the number of color change holes, which represents the number of carbon source used by the microbial community; Average well color development (AWCD), representing the overall carbon substrate utilization potential of cultural microbial communities across all wells per plate.

In order to investigate the aggregate structure, all bulk clod samples from each plot were carefully mixed and then gently sieved to pass through a 10-mm sieve. According to the wet-sieving and dry-sieving protocol, the tested soil was fractionated into > 5, 2 ~ 5, 1 ~ 2, 0.25 ~ 1 and < 0.25 mm aggregates, respectively. All separated aggregates were dried in oven at 60 °C for determining their properties. Macroaggregate content (R), average weight diameter (MWD) and geometric mean diameter (GMD) were calculated by the following equations:

$$ {\text{DR}}_{{0.25}}  = \left( {{\text{WR}}_{{0.25}} } \right) = \frac{{\sum\nolimits_{{i = 1}}^{n} {\left( {w_{i}  > 0.25} \right)} }}{{\sum\nolimits_{{i = 1}}^{n} {(w_{i} )} }} \times 100\% $$

$$ {\text{D - MWD}}\left( {{\text{W - MWD}}} \right) = \sum\limits_{{i = 1}}^{n} {(\bar{d}_{i} w_{i} )} $$

$$ {\text{D - GMD}}\left( {{\text{W - GMD}}} \right) = \exp \left[ {\frac{{\sum\limits_{{i = 1}}^{n} {m_{i} \ln \bar{d}_{i} } }}{{\sum\limits_{{i = 1}}^{n} {m_{i} } }}} \right] $$

where DR_0.25_ and WR_0.25_ are the proportion of > 0.25 mm soil mechanical-stable aggregates and water-stable aggregates, respectively; D-MWD and W-MWD are the mean weight diameter of mechanical-stable aggregates and water-stable aggregates (mm), respectively; D-GMD and W-GMD are the mean geometric diameter of mechanical-stable aggregates and water-stable aggregates (mm), respectively; *m*_*i*_ is mass in size fraction *i*; and *w*_*i*_ is the proportion (%) of the total sample mass in size fraction *i* and *d*_*i*_ is mean diameter of size fraction *i*.

### Evaluation of soil fertility

#### Grey correlation analysis

Grey correlation analysis refers to a method of quantitative description and comparison of a system's development and change. The basic idea is to determine whether they are closely connected by determining the geometric similarity of the reference data column and several comparison data columns, which reflects the degree of correlation between the curves^29^. The grey relational coefficient *ξ*_i_ (*k*) can be expressed as follows:

$$ \xi (k) = \frac{{\mathop {\min }\limits_{i} \mathop {\min }\limits_{k} \left| {x_{0} (k) - x_{i} (k)} \right| + \rho \mathop {\max }\limits_{i} \mathop {\max }\limits_{k} \left| {x_{0} (k) - x_{i} (k)} \right|}}{{\left| {x_{0} (k) - x_{i} (k)} \right| + \rho \mathop {\max }\limits_{i} \max \left| {\mathop {x_{0} (k)}\limits_{k}  - x_{i} (k)} \right|}} $$

$$ x_{i}^{k}  = \frac{{x_{i}^{k} }}{{\mathop {\max }\limits_{i} x_{i}^{k} }} $$

$$ \gamma _{i}  = \frac{1}{n}\sum\limits_{{k = i}}^{n} {\xi _{i} } (k) $$

$$ \omega _{{i(\gamma )}}  = \frac{1}{n}\sum\limits_{{i = 1}}^{n} {\gamma _{i} } $$

$$ G_{i}^{k}  = \sum\limits_{{i = 1}}^{n} {\left( {\xi _{i}  \times \omega _{{i(\gamma )}} } \right),\quad k = 1,2,3, \ldots ,n;\quad i = 1,2,3, \ldots ,n} $$

where $x_{i}^{k}$ The *i* trait observation value of treatment *k*; $\mathop {\max }\limits_{i} x_{i}^{k}$ The maximum value of the *i* trait in all treatments; $\mathop {\min }\limits_{i} x_{i}^{k}$ The minimum value of the *i* trait in all treatments; $\mathop {\min }\limits_{i} \mathop {\min }\limits_{k} \left| {x_{0} (k) - x_{i} (k)} \right|$ Second level minimum difference; $\mathop {\max }\limits_{i} \mathop {\max }\limits_{k} \left| {x_{0} (k) - x_{i} (k)} \right|$ Second level maximum difference; $\rho$ Resolution coefficient (0.5).

#### Principal component analysis

Principal component analysis refers to a multivariate statistical method that converts multiple indicators into several comprehensive indicators by the idea of dimensionality under the premise of losing little information. It simplifies the complexity in high-dimensional data while retaining trends and patterns^30^.

#### Cluster analysis

Cluster analysis comprises a range of methods for classifying multivariate data into subgroups. Using the euclidean distance as a measure of the difference in the fertility of each treatment, the shortest distance method was used to systematically cluster according to the degree of intimacy and similarity of soil fertility levels. By organizing multivariate data into such subgroups, clustering can help reveal the characteristics of any structure or patterns present^31^.

### Statistical analysis

Correlation analysis was performed to assess the relationships between rapeseed yield and soil attributes. Grey correlation analysis and principal component analysis were performed to establish comprehensive score for soil fertility and the main soil factors affecting rapeseed yield. Cluster analysis was used to cluster the soil fertility of each treatment. All the statistical analyses were performed using Excel 2018 (Office Software, Inc., Beijing, China) and SPSS 17.0 (SPSS Inc., Chicago, Illinois, USA). The comparisons of treatment means were based on LSD test at the *P* < 0.05 probability level.

### Ethics statement

Identifies Southwest University that approved the collection of plant or seed specimens.

Confirms that all methods were carried out in accordance with relevant guidelines and regulations.

## Results

### Selection of evaluation indexes for soil fertilization

To comprehensively and objectively evaluate the impact of biochar combined with nitrogen reduction on soil fertility, and identify the main soil environmental driving factors affecting the high yield of rapeseed, following the principles of representativeness, stability, and comparability, 34 indicators from three perspectives of soil physics, chemistry and biology, which represented the status of soil fertility were selected (Table 1)^22–23^. It can be seen that there were significant differences in various indexes of different treatments (except for TN, TP, TK and AN), which could be used to distinguish the fertility effect of different treatments. Then, correlation analysis was performed between the 34 selected indicators and rapeseed yield, and 27 indicators (*x*_1~_*x*_27_) with significant correlations with yield were selected as evaluation indicators based on science and rationalization (Table 2).

**Table 1:** Soil physical, chemical and biological indicators.

Index	N100B0	N100B10	N100B20	N100B40	N80B0	N80B10	N80B20	N80B40	N60B0	N60B10	N60B20	N60B40
ST	19.11bc	19.65a	19.42abc	19.39abc	19.07c	19.54a	19.34abc	19.31abc	19.14bc	19.51a	19.46ab	19.34abc
SWC	0.134ab	0.143a	0.138ab	0.136ab	0.133ab	0.143a	0.137ab	0.134ab	0.132b	0.143a	0.138ab	0.134ab
SOM	14.31b	14.69ab	15.06ab	16.30a	13.79b	14.36b	14.59b	15.18ab	13.71b	14.16b	14.49b	15.01ab
TN	1.12cd	1.27a	1.25ab	1.23abc	1.11cd	1.21abc	1.15abc	1.15abc	1.02d	1.17abc	1.14bcd	1.12cd
TP	0.61a	0.65a	0.65a	0.64a	0.60a	0.65a	0.65a	0.63a	0.59a	0.66a	0.65a	0.65a
TK	18.23a	20.38a	19.81a	19.49a	18.16a	20.48a	19.55a	19.08a	17.95a	20.58a	19.15a	18.99a
AN	42.00a	44.10a	43.00a	42.77a	41.80a	43.07a	42.40a	42.30a	41.67a	42.90a	42.07a	42.00a
AP	12.48bcd	15.66a	14.79ab	13.81abcd	11.27cd	14.88ab	13.57abcd	11.57cd	10.91d	14.27abc	12.85abcd	11.33cd
AK	80.58c	90.09a	83.51bc	83.00bc	80.98c	88.00ab	83.06bc	82.98bc	79.18c	87.10ab	83.92bc	82.96bc
DR_0.25_	87.93ab	91.38a	87.55ab	87.16ab	87.83ab	91.42a	87.46ab	87.01ab	86.85ab	91.11a	85.38b	85.11b
WR_0.25_	66.05b	76.52a	69.90ab	68.42b	65.85b	75.68a	69.63ab	66.36b	65.52b	70.47ab	66.37b	65.93b
D-MWD	4.97abc	5.09a	4.83abc	4.86abc	4.87abc	5.04abc	4.89abc	4.83abc	4.93abc	5.08ab	4.81bc	4.77c
W-MWD	2.40bc	2.81a	2.66abc	2.69abc	2.42bc	2.78a	2.70abc	2.69abc	2.39c	2.57abc	2.65abc	2.75ab
D-GMD	3.03abc	3.38a	2.91abc	2.89abc	2.96abc	3.33ab	2.92abc	2.86bc	2.95abc	3.35ab	2.76c	2.71c
W-GMD	0.90c	1.28a	1.04bc	1.04bc	0.91c	1.24ab	1.06bc	0.98c	0.89c	1.04bc	0.98c	0.99c
POC	2.90cdef	3.38abcd	3.46abc	3.79a	2.88def	3.11bcdef	3.28abcd	3.67ab	2.57f.	2.70ef	3.26abcde	3.61ab
DOC	0.66ab	0.75a	0.71a	0.63ab	0.66ab	0.71a	0.71a	0.56b	0.64ab	0.66ab	0.62ab	0.55b
AOC	8.00ab	8.80a	7.50bc	7.33bc	7.27bc	7.50bc	7.27bc	6.53c	6.77c	7.30bc	7.07bc	6.30c
SUC	40.58ab	41.90a	39.47abcd	38.61bcde	39.44abcd	39.70abc	37.84bcde	37.55cde	38.55bcde	39.58abcd	36.91de	36.39e
URE	2.12bc	2.39a	2.21b	2.05cd	1.51fg	1.92d	1.73e	1.64ef	1.49g	1.76e	1.65ef	1.45g
B	3.13ab	3.86a	3.46ab	2.98ab	2.83b	3.94a	3.43ab	2.97ab	2.82b	3.91a	3.12ab	2.63b
F	0.41abc	0.45ab	0.41abc	0.36bcd	0.36bcd	0.48a	0.35cd	0.35cd	0.29d	0.41abc	0.35cd	0.28d
A	0.05cd	0.27a	0.06c	0.05cd	0.04cd	0.26a	0.05cd	0.04d	0.04d	0.23b	0.04cd	0.03d
GP	1.19abc	1.27ab	1.11abc	1.04bc	1.04bc	1.43a	1.22abc	0.95bc	1.00bc	1.22abc	1.07bc	0.88c
GN	1.09abc	1.32ab	1.20abc	1.05bc	1.00bc	1.31ab	1.29ab	1.08abc	1.04bc	1.43a	1.06bc	0.93c
S	1.16c	1.39a	1.39a	1.28b	1.09d	1.29b	1.17c	1.15c	1.08d	1.26b	1.17c	1.13cd
AA	1.10def	1.38a	1.37a	1.13cde	1.05f.	1.24b	1.15cd	1.07ef	1.04f.	1.21bc	1.14cde	1.06f
PA	0.61abc	0.65a	0.64ab	0.62abc	0.60abc	0.64ab	0.61abc	0.58b	0.57c	0.62abc	0.60abc	0.56c
CA	0.93de	1.19a	1.18a	0.98cd	0.87e	1.10b	1.01c	0.92de	0.87e	1.06bc	0.99cd	0.92de
AM	0.95c	1.07ab	1.08a	0.93c	0.88c	0.97bc	0.94c	0.87c	0.89c	0.95c	0.93c	0.89c
P	1.02ef	1.45a	1.44a	1.06de	0.88g	1.34b	1.13cd	1.05de	0.86g	1.16c	1.00ef	0.97f
H	3.314bcd	3.339a	3.339a	3.304d	3.306d	3.341a	3.325abc	3.309cd	3.307d	3.329ab	3.314bcd	3.297d
D	0.962ab	0.963a	0.963a	0.961d	0.961cd	0.963ab	0.962bc	0.961cd	0.961cd	0.962ab	0.961cd	0.960d
E	0.965bc	0.972a	0.972a	0.962c	0.963c	0.973a	0.968ab	0.964c	0.963c	0.969ab	0.965bc	0.960c

ST/°C; SWC/%; SOM, TN, TP, TK, POC, AOC/g·kg^−1^; AN, AP, AK/mg·kg^−1^; DR_0.25_, WR_0.25_/%; DOC/g·L^−1^; SUC, URE/mg·g^−1^; B, F, A, GP, GN/nmol·g^−1^.

**Table 2:** Correlation coefficients of soil environment factors and rapeseed yield.

Soil physical index	Yield (*x*_0_)	Soil chemical index	Yield (*x*_0_)	Soil biological index	Yield (*x*_0_)
ST	0.285	POC	0.161	B (*x*_15_)	0.339*
SWC (*x*_1_)	0.330*	DOC (*x*_11_)	0.452**	F (*x*_16_)	0.527**
SOM	0.255	AOC (*x*_12_)	0.527**	A (*x*_17_)	0.484**
TN (*x*_2_)	0.640**	SUC (*x*_13_)	0.542**	GP (*x*_18_)	0.346*
TP	-0.001	URE (*x*_14_)	0.814**	GN	0.269
TK	0.318			S (*x*_19_)	0.797**
AN (*x*_3_)	0.373*			AA (*x*_20_)	0.684**
AP (*x*_4_)	0.573**			PA (*x*_21_)	0.634**
AK (*x*_5_)	0.433**			CA (*x*_22_)	0.658**
DR_0.25_ (*x*_6_)	0.476**			AM (*x*_23_)	0.519**
WR_0.25_ (*x*_7_)	0.606**			P (*x*_24_)	0.759**
D-MWD (*x*_8_)	0.343*			H (*x*_25_)	0.594**
W-MWD	0.327			D (*x*_26_)	0.580**
D-GMD (*x*_9_)	0.429**			E (*x*_27_)	0.590**
W-GMD (*x*_10_)	0.570**				

*Significant at the 0.05 probability level.

**Significant at the 0.01 probability level.

### Grey correlation analysis

#### Comparison of correlation degree

According to the correlation analysis principle in the grey system theory, the greater the correlation of the evaluation index, the closer it is to the reference index, that is, the greater the weight coefficient of evaluation index, the greater the impact on yield. As could be seen from Table 3, AP (weight coefficient 0.041) and W-GMD (weight coefficient 0.040) had the greatest impact on yield in physical index, and URE (weight coefficient 0.040) of soil chemical indicators had the greatest impact on yield. Besides, in biological index, F (weight coefficient 0.040), S (weight coefficient 0.040), AA (weight coefficient 0.040), P (weight coefficient 0.040) and CA (weight coefficient 0.040) by microorganisms had the greatest impact on yield. These indicators could be used as the main soil factors affecting rapeseed yield under biochar combined with nitrogen reduction.

**Table 3:** Correlation coefficient.

Index	N100B0	N100B10	N100B20	N100B40	N80 B0	N80 B10	N80 B20	N80 B40	N60 B0	N60 B10	N60 B20	N60 B40	*γ*_i_	*w*_i_
*x*_1_	0.767	0.997	0.982	0.982	0.647	0.898	0.750	0.739	0.580	0.610	0.524	0.495	0.748	0.035
*x*_2_	0.858	1.000	0.933	0.931	0.717	0.998	0.833	0.783	0.707	0.694	0.574	0.531	0.796	0.038
*x*_3_	0.744	1.000	0.963	0.929	0.630	0.941	0.745	0.705	0.559	0.637	0.530	0.484	0.739	0.035
*x*_4_	0.942	1.000	0.959	0.868	0.993	0.993	0.913	0.865	0.875	0.710	0.651	0.681	0.871	0.041
*x*_5_	0.838	1.000	0.920	0.953	0.685	0.940	0.807	0.758	0.618	0.644	0.547	0.504	0.768	0.036
*x*_6_	0.731	0.999	0.993	0.967	0.617	0.890	0.752	0.715	0.555	0.613	0.545	0.498	0.740	0.035
*x*_7_	0.900	1.000	0.891	0.892	0.735	0.913	0.828	0.849	0.642	0.697	0.602	0.547	0.791	0.037
*x*_8_	0.712	1.000	0.970	0.963	0.620	0.910	0.747	0.720	0.539	0.612	0.537	0.494	0.735	0.035
*x*_9_	0.835	1.000	0.793	0.820	0.716	0.919	0.917	0.891	0.623	0.618	0.654	0.597	0.782	0.037
*x*_10_	0.770	1.000	0.725	0.745	0.964	0.959	0.999	0.922	0.884	0.862	0.720	0.625	0.848	0.040
*x*_11_	0.862	1.000	0.961	0.784	0.714	0.980	0.766	0.872	0.649	0.759	0.641	0.676	0.805	0.038
*x*_12_	0.812	1.000	0.780	0.782	0.788	0.793	0.989	0.871	0.751	0.836	0.670	0.691	0.814	0.038
*x*_13_	0.721	1.000	0.954	0.953	0.637	0.987	0.840	0.797	0.580	0.669	0.590	0.542	0.773	0.037
*x*_14_	0.849	1.000	0.913	0.822	0.810	0.719	0.786	0.776	0.950	0.953	0.838	0.859	0.856	0.040
*x*_15_	0.936	0.949	0.824	0.675	0.989	0.890	0.903	0.895	0.837	0.617	0.684	0.757	0.830	0.039
*x*_16_	0.942	0.850	0.778	0.657	0.952	0.890	0.791	0.837	0.910	0.782	0.756	0.892	0.837	0.040
*x*_17_	0.376	1.000	0.339	0.333	0.403	0.932	0.374	0.368	0.440	0.789	0.459	0.479	0.524	0.025
*x*_18_	0.971	0.775	0.678	0.646	0.990	0.890	0.949	0.741	0.875	0.799	0.742	0.844	0.825	0.039
*x*_19_	0.959	0.999	0.906	0.956	0.865	0.946	0.977	0.932	0.740	0.720	0.632	0.591	0.852	0.040
*x*_20_	0.942	1.000	0.916	0.761	0.909	0.873	0.993	0.942	0.771	0.763	0.641	0.633	0.845	0.040
*x*_21_	0.761	1.000	0.932	0.971	0.659	0.916	0.772	0.798	0.622	0.663	0.548	0.544	0.766	0.036
*x*_22_	0.907	1.000	0.917	0.768	0.976	0.932	0.951	0.944	0.812	0.743	0.635	0.625	0.851	0.040
*x*_23_	0.863	0.980	0.906	0.825	0.799	0.874	0.915	0.982	0.679	0.761	0.609	0.577	0.814	0.038
*x*_24_	0.771	1.000	0.925	0.644	0.767	0.938	0.885	0.843	0.880	0.893	0.836	0.759	0.845	0.040
*x*_25_	0.691	0.998	0.908	0.888	0.589	0.890	0.699	0.667	0.525	0.613	0.504	0.464	0.703	0.033
*x*_26_	0.683	1.000	0.907	0.870	0.582	0.890	0.694	0.659	0.519	0.610	0.500	0.458	0.698	0.033
*x*_27_	0.691	0.998	0.908	0.888	0.589	0.890	0.699	0.667	0.525	0.613	0.504	0.464	0.703	0.033

#### Comprehensive evaluation and analysis of soil fertilization

Grey comprehensive evaluation values were enumerated in Table 4. According to the result, the variation trend of soil fertility under each treatment was consistent with the change trend of yield. Under the same nitrogen level, the comprehensive evaluation value of B10 was greater than that of B0, B20 and B40 treatment; under the same biochar level, the comprehensive evaluation value of N60 and N80 treatment is less than N100 treatment. Among all the treatments, N100B10 had the highest comprehensive evaluation value, ranking first, followed by N100B20 and N80B10, and N60B40 had the lowest comprehensive evaluation value. Then the conclusion was that B10 was conducive to soil fertilization and crop yield increase, which combined with N80 could achieve nitrogen reduction and efficiency increase, while nitrogen reduction (N60) was not conducive to soil fertilization and crop yield increase.

**Table 4:** Grey judgement analysis of soil fertilization in different treatments.

Treatments	Grey comprehensive evaluation value	Comprehensive ranking	Yield value	Yield ranking
N100B0	0.817	6	2.11	6
N100B10	0.980	1	2.57	1
N100B20	0.877	3	2.47	2
N100B40	0.825	5	2.42	4
N80B0	0.764	8	1.86	9
N80B10	0.909	2	2.45	3
N80B20	0.834	4	2.13	5
N80B40	0.806	7	2.05	7
N60B0	0.700	10	1.65	10
N60B10	0.716	9	1.94	8
N60B20	0.623	11	1.58	11
N60B40	0.611	12	1.40	12

### Principal component analysis

#### Correlation analysis between evaluation indicators

By analyzing related relationship of 27 indicators of soil physics, chemistry, and biology highly correlated with yield (Table 5), significant or extremely significant correlation between the indicators was found, indicating that there was information overlap between indicators.

**Table 5:** Correlation analysis.

	*x*_1_	*x*_2_	*x*_3_	*x*_4_	*x*_5_	*x*_6_	*x*_7_	*x*_8_	*x*_9_	*x*_10_	*x*_11_	*x*_12_	*x*_13_	*x*_14_	*x*_15_	*x*_16_	*x*_17_	*x*_18_	*x*_19_	*x*_20_	*x*_21_	*x*_22_	*x*_23_	*x*_24_	*x*_25_	*x*_26_	*x*_27_
*x*_1_	–	*	**	^ns^	^ns^	*	**	*	*	**	**	^ns^	^ns^	^ns^	^ns^	*	**	^ns^	*	*	^ns^	**	^ns^	**	**	*	*
*x*_2_		–	**	^ns^	**	^ns^	**	^ns^	^ns^	**	^ns^	*	^ns^	**	^ns^	^ns^	*	^ns^	**	**	**	**	^ns^	**	**	**	**
*x*_3_			–	^ns^	*	^ns^	^ns^	^ns^	^ns^	^ns^	^ns^	^ns^	^ns^	^ns^	^ns^	^ns^	^ns^	^ns^	*	^ns^	*	*	^ns^	*	^ns^	^ns^	^ns^
*x*_4_				–	*	*	**	^ns^	**	**	^ns^	**	*	**	*	**	**	^ns^	**	**	*	**	**	**	**	**	**
*x*_5_					–	*	**	^ns^	*	**	^ns^	*	^ns^	^ns^	^ns^	**	**	^ns^	**	**	**	**	*	**	**	**	**
*x*_6_						–	**	**	**	**	*	*	*	*	**	**	**	**	**	**	*	**	*	**	**	**	**
*x*_7_							–	**	**	**	**	**	^ns^	**	**	**	**	**	**	**	**	**	**	**	**	**	**
*x*_8_								–	**	^ns^	^ns^	**	**	*	^ns^	*	**	^ns^	^ns^	^ns^	*	*	^ns^	*	**	**	**
*x*_9_									–	**	*	**	*	*	**	**	**	**	*	**	*	**	*	**	**	**	**
*x*_10_										–	*	*	^ns^	**	**	*	**	*	**	**	*	**	**	**	**	**	**
*x*_11_											–	^ns^	^ns^	**	**	**	*	*	*	*	^ns^	**	*	*	*	**	*
*x*_12_												–	**	**	^ns^	^ns^	*	^ns^	**	**	**	**	**	**	**	**	**
*x*_13_													–	**	^ns^	*	**	^ns^	**	**	^ns^	*	*	*	*	**	*
*x*_14_														–	*	**	**	^ns^	**	**	**	**	**	**	**	**	**
*x*_15_															–	**	**	**	**	**	^ns^	**	*	**	**	**	**
*x*_16_																–	**	**	**	**	*	**	**	**	**	**	**
*x*_17_																	–	**	**	**	**	**	*	**	**	**	**
*x*_18_																		–	^ns^	*	^ns^	*	^ns^	**	*	**	*
*x*_19_																			–	**	**	**	**	**	**	**	**
*x*_20_																				–	**	**	**	**	**	**	**
*x*_21_																					–	**	**	**	**	**	**
*x*_22_																						–	**	**	**	**	**
*x*_23_																							–	**	**	**	**
*x*_24_																								–	**	**	**
*x*_25_																									–	**	**
*x*_26_																										–	**
*x*_27_																											–

*Significant at the 0.05 probability level.

**Significant at the 0.01 probability level; ns Not significant.

#### Calculation of feature vector

The feature values and contribution rates of each principal component were shown in Table 6 based on the descending dimension algorithm of 27 indicators (principal components with specified feature values greater than 1 were extracted). The eigenvalues of three principal components were greater than 1 with eigenvalues of 20.496, 2.487, and 1.708, and contribution rates of 75.911%, 9.211%, and 6.327%, respectively. The cumulative contribution rate of the first three principal components reached 91.448%, which could reflect enough information. Therefore, three main components were selected to comprehensively analyze and evaluate various indicators of soil environment under biochar combined with nitrogen reduction.

**Table 6:** Eigenvalues and cumulative contribution proportions of principle components of the indices.

Principal component	Eigenvalue	Contribution rate (%)	Accumulative contribution rate (%)
1	20.496	75.911	75.911
2	2.487	9.211	85.122
3	1.708	6.327	91.448

A component matrix (the data was not listed in this paper) could be obtained by the descending dimension algorithm, and feature vector could be calculated by each value in the component matrix (components 1, 2, and 3) dividing by the square root of the corresponding eigenvalue (Table 7). In the main component 1, the variance of AP (feature vector 0.210) and W-GMD (feature vector 0.208) in the soil physical indicators, DOC (feature vector 0.181) in the soil chemical indicators, and B (feature vector 0.206), F (feature vector 0.201), S (feature vector 0.195), AA (feature vector 0.201), PA (feature vector 0.206), P (feature vector 0.204), CA (feature vector 0.203), H (feature vector 0.206), D (feature vector 0.204) and E (feature vector 0.205) in the soil biological indicators was the largest. Principal component 1 explained 75.911% of the difference, which represented most of the indicator information. Therefore, The indicator that contributed to the variance of principal component 1 could not only be used as the main factor affecting soil fertility but also be used as the main factor affecting rape yield under different treatments.

**Table 7:** Eigenvalues of the indices under the three principal components.

Index	Prin1	Prin2	Prin3
*x*_1_	0.197	0.053	− 0.285
*x*_2_	0.176	− 0.307	− 0.093
*x*_3_	0.203	− 0.114	− 0.133
*x*_4_	0.210	− 0.129	− 0.056
*x*_5_	0.186	0.019	− 0.371
*x*_6_	0.187	0.306	− 0.033
*x*_7_	0.208	0.047	− 0.168
*x*_8_	0.160	0.409	0.047
*x*_9_	0.180	0.349	− 0.018
*x*_10_	0.191	− 0.036	− 0.286
*x*_11_	0.181	0.060	0.298
*x*_12_	0.176	0.048	0.362
*x*_13_	0.163	0.189	0.393
*x*_14_	0.176	− 0.193	0.294
*x*_15_	0.206	0.123	− 0.112
*x*_16_	0.201	0.093	0.065
*x*_17_	0.187	0.254	− 0.233
*x*_18_	0.188	0.216	0.056
*x*_19_	0.195	− 0.254	− 0.017
*x*_20_	0.201	− 0.223	0.021
*x*_21_	0.206	− 0.096	0.140
*x*_22_	0.203	− 0.222	− 0.077
*x*_23_	0.187	− 0.249	0.191
*x*_24_	0.204	− 0.198	− 0.057
*x*_25_	0.206	− 0.007	0.010
*x*_26_	0.204	0.006	0.191
*x*_27_	0.205	0.001	0.009

#### Comprehensive evaluation and analysis of soil fertilization

In order to evaluate the fertilization effect of each treatment intuitively and accurately, the corresponding Y value was calculated according to the feature vector and the standardized value: Y1 = 0.197*x*_1_ + 0.176*x*_2_ + 0.203*x*_3_ + 0.210*x*_4_ + 0.186*x*_5_ + 0.187*x*_6_ + 0.208*x*_7_ + 0.160*x*_8_ + 0.180*x*_9_ + 0.191*x*_10_ + 0.181*x*_11_ + 0.176*x*_12_ + 0.163*x*_13_ + 0.176*x*_14_ + 0.206*x*_15_ + 0.201*x*_16_ + 0.187*x*_17_ + 0.188*x*_18_ + 0.195*x*_19_ + 0.201*x*_20_ + 0.206*x*_21_ + 0.203*x*_22_ + 0.187*x*_23_ + 0.204*x*_24_ + 0.206*x*_25_ + 0.204*x*_26_ + 0.205*x*_27_. Similarly, Y2 and Y3 were also calculated. The weighted mean was obtained by taking the contribution rate of the three principal components as the weight, and the composite score of principal component Y = 75.911%Y1 + 9.211%Y2 + 6.327%Y3. The comprehensive score could be used as a comprehensive evaluation index for the effect of biochar combined with nitrogen reduction on soil fertility (Table 8). It could be seen from Table 8, the change trend of soil fertilization in each treatment was consistent with that of yield. Under the same nitrogen application rate, the comprehensive score of B10 treatment was greater than that of B0, B20, and B40 treatment; under the same biochar, the comprehensive score of N60 and N80 treatment was less than that of N100 treatment. Among all the treatments, N100B10 had the highest comprehensive score, followed by N80B10, and N60B40 was lowest. It indicated that B10 was conducive to soil fertilization and crop yield increase, and the combined with N80 could achieve nitrogen reduction and efficiency increase, while nitrogen reduction (N60) was not conducive to soil fertilization and crop yield increase.

**Table 8:** Principle analysis of soil fertilization in different treatments.

Treatments	Comprehensive value	Comprehensive ranking	Yield value	Yield ranking
N100B0	− 0.326	6	2.11	6
N100B10	6.519	1	2.57	1
N100B20	2.683	3	2.47	2
N100B40	− 0.817	7	2.42	4
N80B0	− 2.541	9	1.86	9
N80B10	4.692	2	2.45	3
N80B20	0.053	5	2.13	5
N80B40	− 2.802	10	2.05	7
N60B0	− 3.547	11	1.65	10
N60B10	2.540	4	1.94	8
N60B20	− 1.893	8	1.58	11
N60B40	− 4.560	12	1.40	12

### Cluster analysis

**Figure 1:**
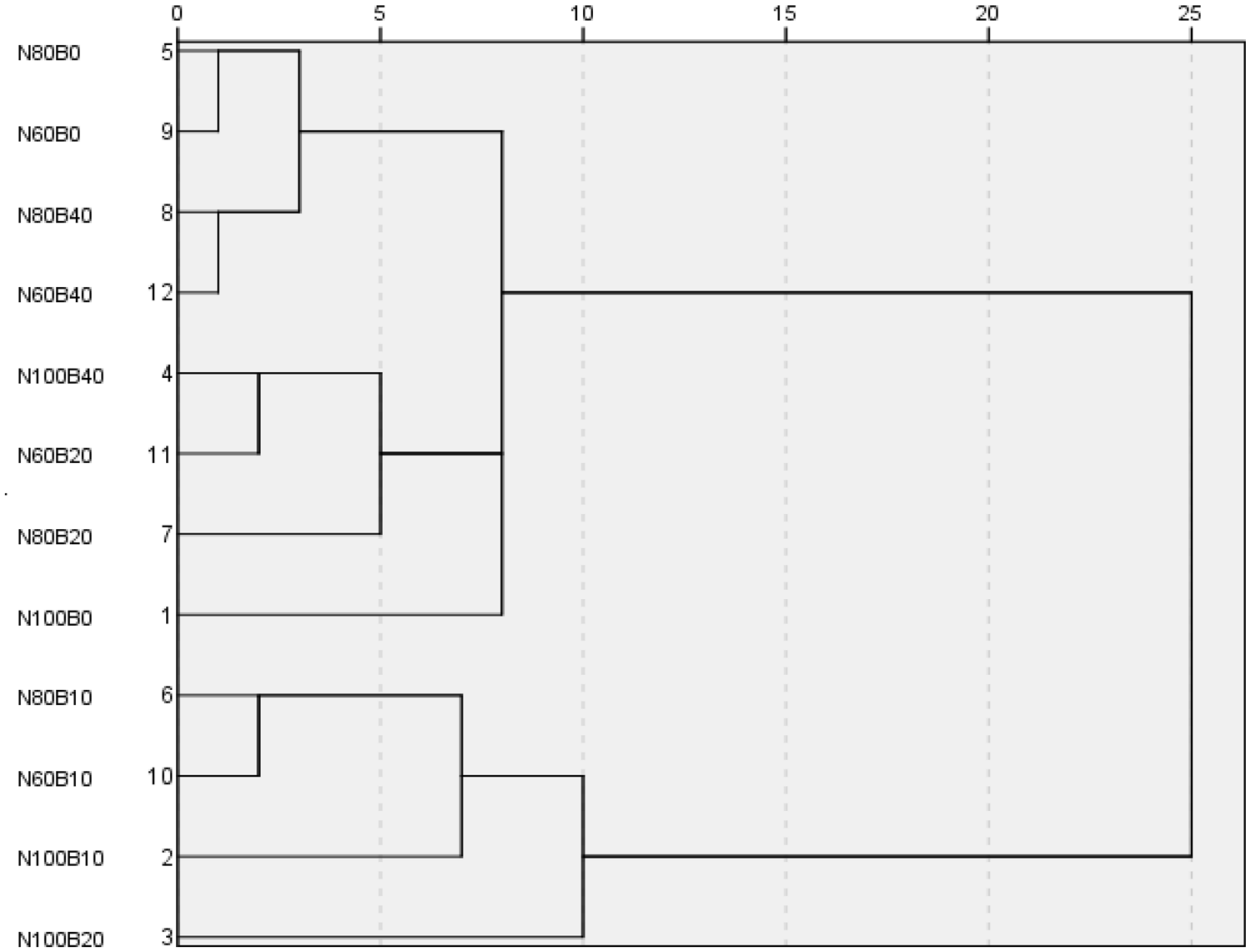
Cluster analysis of soil fertilization in each treatment.

As shown in Fig. 1, if the distance threshold was set to 8, the systematic clustering according to the degree of intimacy and similarity of the soil fertility level could well reflect the effect of biochar combined with nitrogen reduction on soil fertility. In short, the 12 treatments could be roughly divided into 5 categories. In other words, N80B0, N80B40, N60B0 and N60B40 belonged to a category, which were considered a low fertility level; N100B10, N80B10 and N60B10 belonged to a category, which were considered a high fertility level; N100B40, N60B20 and N80B20 belonged to a category, and N100B0 and N100B20 was each a category, which were considered a medium fertility levels. It showed that biochar and nitrogen fertilizer could affect the soil fertility level in different degrees.

## Discussion

Improving soil fertility requires not only building a good soil structure, but also cultivating fertile farming layers to improve soil productivity. Therefore, the evaluation of soil fertilization in each treatment must be comprehensively reflected from the perspectives of soil physics, chemistry and biology^32^. In this study, 27 indicators being significantly correlated with yield were selected as evaluation indicators through correlation analysis. Correlation analysis showed that there were different degrees of correlation between soil physical, chemical and biological indicators, indicating that they were connected and interact with each other, and they jointly affected the soil fertilization effect, which was similar to the results of Yuan et al.^33^. However, the selection of evaluation indicators and evaluation methods is slightly different due to different test purposes and there is no uniform evaluation standard and fixed method in the world, but evaluation indicators generally cover soil physical, chemical and biological indicators and evaluation methods generally include grey correlation analysis, path analysis, principal component analysis, factor analysis and cluster analysis. In our experiment, gray correlation analysis and principal component analysis showed that the comprehensive evaluation value and comprehensive score were basically consistent with rapeseed yield. Generally, the features of soil quality can often be directly and comprehensively displayed through crop yields, making the comprehensive evaluation results more accurate, objective and scientific^34^. Therefore, the selected indicators and methods in this study were reliable for evaluating soil fertility status.

Our study results indicated that biochar combined with nitrogen reduction mainly changed AP and W-GMD in soil physical indexes, URE in soil chemical indicators, and F, S, AA, P and CA in soil biological indexes. This may be related to the local low-phosphorus and arid environment. Currently, there are few studies on the evaluation of soil quality under biochar combined with nitrogen reduction, and further research verification are needed. From the feature vector of the first principal component of principal component analysis, it could be seen that biochar combined with nitrogen reduction mainly changed AP and W-GMD in soil physical indicators, DOC in soil chemical indicators, and B, S, AA, PA, P, CA, H, D and E in soil biological indicators. Principal component 1 explained 75.911% of the difference, which basically reflected the information provided by all soil indicators, making the evaluation more scientific and reasonable. Therefore, we summarized that the available phosphorus, the geometric mean diameter of water stability, fungi number, the utilization degree of microorganisms on sugars, amino acids, polymers and carboxylic acids could be used as the main soil factors affecting rapeseed yield under biochar combined with nitrogen reduction.

Cluster analysis showed that no biochar, nitrogen fertilizer reduction and high biochar resulted in low soil fertility levels, while appropriate biochar, moderate nitrogen fertilizer, and biochar combined with nitrogen reduction brought about high soil fertility levels. This indicated that appropriate amount of biochar and nitrogen fertilizer was beneficial to the improvement of soil fertility, being similar to reports by Nasim et al.^35^ and Veysel et al.^36^. This was mainly because biochar itself provides nutrients and retains nutrients, and changes the kinetics of soil microorganisms, thus promoting biological carbon fixation^37^. As for nitrogen fertilizer, it is a kind of quick-acting nitrogen, which is beneficial to the improvement of soil effective nutrients after applied to the soil^38^. Generally, the yield can reflect the soil fertility to a certain extent. In this study, rapeseed yields of N100B10, N100B20 and N80B10 were the highest, followed by other treatments at the N100 and B10 levels, while the yields at the N60, B40 and B0 levels were the lowest, being basically consistent with the results of cluster analysis. It was feasible to use cluster analysis to classify the soil fertility level, which was in line with objective reality, and could be used as a basis for evaluating the effect of biochar combined with nitrogen reduction on soil fertility.

## Conclusions

Available phosphorus, geometric mean diameter of water stability, fungi number, utilization of microorganisms on sugars, amino acids, polymers and carboxylic acids were the main soil factors affecting soil fertilization and rapeseed yield under biochar combined with nitrogen reduction based on grey correlation analysis and principal component analysis. Besides, based on grey correlation analysis, principal component analysis and cluster analysis, the combined application of 10 t hm^−2^ biochar and 144 or 180 kg hm^−2^ nitrogen fertilizers had better fertilization effect. From the perspective of comprehensive economic and environmental benefits, 10 t hm^−2^ biochar combined with 144 kg hm^−2^ nitrogen fertilizer was the optimal fertilization model in uplands in purple soil area of southwest China.
